# Marginal Fit, Mechanical Properties, and Esthetic Outcomes of CAD/CAM Interim Fixed Dental Prostheses (FDPs): A Systematic Review

**DOI:** 10.3390/ma16051996

**Published:** 2023-02-28

**Authors:** Hussain Al-humood, Amal Alfaraj, Chao-Chieh Yang, John Levon, Tien-Min Gabriel Chu, Wei-Shao Lin

**Affiliations:** School of Dentistry, Indiana University, Indianapolis, IN 46202, USA

**Keywords:** 3D-printed, milled, strength, color stability, marginal gap

## Abstract

This systematic review aimed to study the outcomes of CAD-CAM (milled and 3D-printed) interim dental prostheses when compared to conventional ones. The focused question of “In natural teeth, what are the outcomes of CAD-CAM interim FDPs compared to the conventionally-manufactured ones regarding marginal fit, mechanical properties, esthetics, and color stability” was formulated. The systematic search was conducted electronically in the PubMed/MEDLINE, CENTRAL, EMBASE, Web of Science, New York Academy of Medicine Grey Literature Report, and Google Scholar databases by using the MeSH keywords and keywords associated with the focused question and limiting articles to those published between 2000 and 2022. A manual search was conducted in selected dental journals. The results were analyzed qualitatively and are presented in table format. Of the included studies, 18 studies were in vitro and 1 was a randomized clinical trial. Of the eight studies analyzing the mechanical properties, five studies favored the milled interim restorations, one study favored both 3D-printed and milled interim restorations, and two studies reported better mechanical properties in conventional interim restorations. Among four studies evaluating the marginal discrepancies, two studies favored the marginal fit in milled interim restorations, one study reported a better marginal fit in both milled and 3D-printed interim restorations, and one study found conventional interim restorations have a better marginal fit and smaller marginal discrepancy when compared to both milled and 3D-printed restorations. Among five studies that evaluated both the mechanical properties and marginal fit, 1 study favored 3D-printed interim restorations and four studies favored milled interim restorations over the conventional ones. Two studies analyzing the esthetics outcomes demonstrated better results with milled interim restorations compared to conventional and 3D-printed interim restorations in terms of their color stabilities. The risk of bias was low for all the studies reviewed. The high level of heterogeneity within the studies excluded meta-analysis. Most of the studies favored the milled interim restorations over the 3D-printed and conventional restorations. The results suggested that milled interim restorations offer a better marginal fit, higher mechanical properties, and better esthetic outcomes in terms of color stabilities.

## 1. Introduction

A provisional or interim prosthesis replicates the planned definitive prosthesis in form and function, which helps in assessing the effectiveness of the planned treatment [1]. These restorations protect teeth from pulpal injury due to thermal, mechanical, or physical stimulus and reduce bacterial contamination, promoting soft tissue healing [2,3]. Conventionally, polymethyl methacrylate (PMMA) and bis-acryl resins are often used to fabricate provisional fixed dental prostheses [4]. These provisional fixed restorations can be fabricated directly on the prepared teeth or indirectly in a dental laboratory [5]. Because of its low polymerization shrinkage and better color stability and mechanical properties, the bis-acryl resin is considered a good alternative to the PMMA [2,6,7]. However, studies suggest that the flexural strength of bis-acryl resin is lower compared to PMMA and the cost is relatively high [2,5]. Conventional interim restorations are prepared manually either directly from a prefabricated template that is filled with resins and placed over the prepared teeth or indirectly by taking an impression of prepared teeth [8,9,10]. Although these chairside manual preparations are more convenient, they may cause air entrapment into resins during mixing procedures. This gives rise to voids and porosity, which can affect the surfactant texture, mechanical properties, longevity, and fit of the restorations [11,12].

The introduction of computer-aided design and computer-aided manufacturing (CAD-CAM) technology in the 1970s led to a rise in its popularity in dentistry. The development of CAD-CAM technology in dentistry has facilitated the overcoming of several disadvantages of conventional methods in the fabrication of both provisional and definitive restorations. The CAD-CAM provisional prostheses can be subtractively manufactured from the milling of prepolymerized acrylic blocks, and these milled prostheses have a high strength and marginal accuracy along with good color stability [2,3,6,7]. In addition, additive manufacturing or 3D printing can also be used to manufacture CAD-CAM provisional prostheses. Based on the recent “ISO/ASTM 52900 Standard Terminology for Additive Manufacturing—General Principles—Terminology”, vat photopolymerization technologies including stereolithography (SLA), digital light processing (DLP), and continuous liquid interface production (CLIP) are commonly used in the fabrication of 3D-printed provisional restorations [13,14]. Although 3D-printing technologies manufacture prostheses with superior details and smooth surfaces, the limited availabilities of applicable materials and published studies are the major challenges [13,14,15,16]. Milled interim restorations could offer higher mechanical properties (flexural strength and fracture toughness), better esthetic outcomes [17,18,19,20,21], and superior marginal fit [22] when compared to traditional restorations.

Using milling or 3D-printing technologies to fabricate interim prostheses can save time, improve patient comfort, and simplify the laboratory process. In case of a fracture of interim prostheses, the digital design file can be used to remanufacture the prostheses with ease [21]. To date, no systematic review was attempted to compare the marginal fit, mechanical properties, and esthetic outcomes of CAD-CAM (both milled and 3D-printed) interim fixed dental prostheses (FDPs) with those of conventional interim prostheses. This systematic review aimed to compare the outcomes of CAD-CAM interim FDP materials to the traditional ones in terms of their marginal fit, mechanical properties, and esthetics.

## 2. Material and Methods

A systematic review of the scientific literature was conducted following the Preferred Reporting of Systematic Reviews and Meta-Analyses (PRISMA) guidelines (Table S1). The review was registered in an international database of prospectively registered systematic reviews (PROSPERO, CRD42022309993; Centre for Reviews and Dissemination, University of York, York, UK). The following focused question was formulated by using the Population, Intervention, Comparison, and Outcome (PICO) format [23]: “In natural teeth, what are the outcomes of CAD-CAM interim FDPs compared to the conventionally-manufactured ones regarding marginal fit, mechanical properties, esthetics, and color stability”. The population was defined as the interim fixed dental prostheses used to restore natural teeth. The intervention was CAD/CAM interim FDPs, which included the milled and 3D-printed interim prostheses. The comparison was interim FDPs fabricated using traditional techniques or materials. Outcomes were the marginal fit, mechanical properties, esthetic outcomes, and color stabilities of the interim FDPs.

The inclusion criteria included in vitro and in vivo studies, studies comparing CAD/CAM interim FDPs to conventional ones, and articles published in English between 2000 and 2022. The exclusion criteria included case reports with less than six patients, review articles, studies without comparative methodology, studies with multiple publications on the same patient population, or publications in languages other than English. The systematic search strategy was conducted electronically in the PubMed/MEDLINE, Cochrane Central Register of Controlled Trials (CENTRAL), EMBASE, and Web of Science databases by using the MeSH keywords and keywords associated with the focused question (Table 1). Grey literature was searched through electronic screening using the New York Academy of Medicine Grey Literature Report (http://greylit.org, accessed on 19 February 2023) and through Google Scholar. All electronic sources were imported into a systematic review management software (Covidence; Melbourne, Australia) to aid in the data collection and extraction. Additionally, the following journals were manually searched for potentially relevant articles: Journal of Prosthetic Dentistry, Journal of Prosthodontics, International Journal of Prosthodontics, Journal of Dentistry, Journal of The American Dental Association, Journal of Operative Dentistry, and Dental Materials. The Cochrane Collaboration tool was used to assess the quality of randomized controlled trials (RCTs) [24], and the Newcastle-Ottawa Scale (NOS) was used to assess the quality of nonrandomized studies [25]. For the data extraction, the following information was subtracted and collected from the selected studies: general study characteristics (authors, publication year, type of study, and sample size), type of interim FDP, material, fabrication method, marginal qualities (gap, fit, integrity, adaptation, misfit, and internal space), mechanical properties (fracture strength, compressive strength, flexural strength, fracture toughness, wear, roughness, elastic modulus, peak stress, and failure load), and esthetic outcomes (translucency parameter). The results were analyzed qualitatively and are presented in table format below.

## 3. Results

### 3.1. Study Screening and Selection

**Figure 1:**
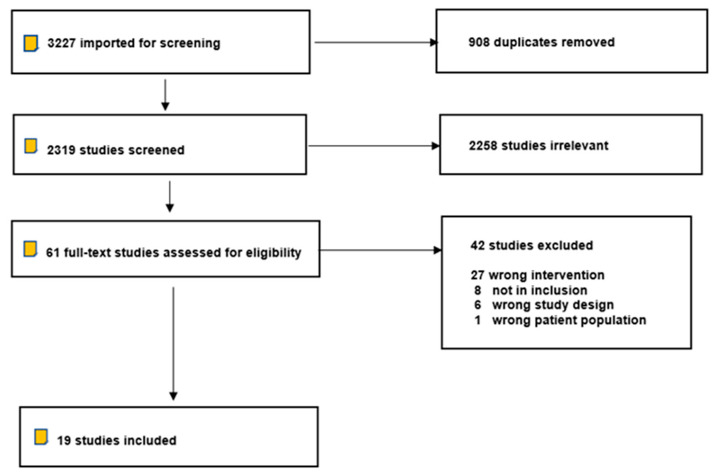
PRISMA flow diagram.

After the initial electronic and manual search, 3227 studies found were found. After removing 908 duplicated studies, 2319 studies were included in the initial screening. After title and abstract screening, 2258 studies were excluded because they did not meet the inclusion criteria of the study. A total number of 61 studies were included in the full-text assessment for eligibility, and 42 studies were excluded (Figure 1). Nineteen studies published between the years 2011 and 2021 were included in the systematic review [2,3,6,26,27,28,29,30,31,32,33,34,35,36,37,38,39,40,41]. All of the articles were assessed and reviewed by two reviewers (H.A. and A.A.) to determine the exclusions and inclusions, and any difference in opinions was resolved based on personal discussion and consensus.

### 3.2. Data Quality Assessment

Out of the 19 included studies, 18 were in vitro and only one study was an RCT. The Newcastle–Ottawa bias assessment tool was used to assess the quality of the nonrandomized studies (Table 2), and the Cochrane bias assessment tool was used to assess bias risk in the RCT (Table 3).

### 3.3. Data Extraction and Analysis

Data extraction and qualitative comparisons were completed for the 19 included studies. The results are summarized in Table 4.

### 3.4. Marginal Fit

Aldahian et al. reported marginal adaptation and marginal discrepancy of 269.94 µm and 395.89 µm, respectively, with the conventional PMMA material; meanwhile, milled and 3D-printed materials demonstrated marginal adaptation and marginal discrepancy of 269.94 µm and 244.95 µm and 197.82 µm and 211.87 µm, respectively. Micro CT was used to measure these two parameters, and measurements were recorded at specific locations for assessments. This study concluded that both milled and 3D-printed materials had better marginal adaptation and fit and minimal discrepancies when compared to conventional ones [28]. In their clinical study, Cheng et al. compared the marginal fit of milled and conventional PMMA materials. The authors reported a marginal fit of 1.15 ± 0.37 μm with milled PMMA material and 1.50 ± 0.69 μm with the conventional PMMA material. The study concluded that milled interim crowns fabricated had a smaller gap and better fit compared to the conventional method [26]. The marginal gap was noted by an independent investigator and assessed using the California Dental Association criteria and the World Dental Federation’s recommendation.

Wu et al. conducted a study to compare the marginal discrepancy in interim crowns fabricated with three different materials—conventional bis-acryl composite resin, milled, and 3D-printed denture teeth resin. Marginal discrepancies were calculated using the polyvinyl siloxane-replica method and optical coherence tomographic scanning. In the measurements of these two methods, the marginal discrepancy of the conventional material was observed to be 71.3 ± 64.9 μm and 82.7 ± 65.8 μm, respectively; the marginal discrepancy of the milled nanoceramic material was 96.9 ± 60.2 μm and 99.6 ± 54.6 μm, respectively; and the marginal discrepancy of the 3D-printed denture teeth was observed to be 120.8 ± 70.9 μm and 143.1 ± 39.9 μm, respectively. This study concluded that the conventionally fabricated provisional crowns using the bis-acryl composite resin material presented a lower gap distance, a better internal fit, and a smaller absolute marginal discrepancy [27]. Angwarawong et al. compared the marginal gap of interim restorations fabricated using conventional materials such as PMMA and bis-acryl resin with that of milled and 3D-printed materials. They measured the marginal gap using a traveling stereomicroscope at eight predetermined points. The marginal gap for each material before and after artificial aging was observed to be: PMMA—85 ± 23 μm (before aging) and 114 ± 29 μm (after aging); bis-acryl—88 ± 17 μm (before aging) and 109 ± 15 μm (after aging); milled material—54 ± 8 μm (before aging) and 74 ± 9 μm (after aging); and 3D-printed material—56 ± 7 μm (before aging) and 71 ± 7 μm (after aging). The authors reported that the milled and 3D-printed groups showed smaller marginal gaps that resulted in better marginal adaptability than the conventional material groups both before and after artificial aging [28].

Al Deeb et al. compared the marginal fit of milled, conventional PMMA, and 3D-printed materials using micro CT. The marginal misfit was reported to be 68.2 ±18.1 μm for the milled material, 283.3 ± 98.6 μm for the conventional PMMA material, and 84.7 ± 27.5 μm for the 3D-printed material. The authors concluded that the milled material showed a superior marginal fit to the conventional material [39]. Abdullah et al. conducted a study in 2018 comparing the marginal fit of three milled and one conventional bis-acryl resin material. They concluded that the milled restorations showed a smaller marginal gap and internal fit with a superior fracture strength in comparison to the conventional samples [40]. The marginal gap and internal fit of the three milled materials and one conventional material were 59.97 ± 11.1 μm and 117.8 ± 15.58 μm; 45.58 ± 9.99 μm and 109.27 ± 19.21 μm; 62.19 ± 12.9 μm and 123.16 ± 23.97 μm; and 138.6 ± 10.1 μm and 140.1 ± 26.53 μm, respectively. Another study by Abdullah et al. assessed the marginal and internal fit of three other milled materials and one bis-acryl resin material. Similar to the previous study, the conventional bis-acryl resin group showed the largest marginal gap value of 193.07 ± 35.96 um and internal fit value of 143.48 ± 26.74 μm. The authors concluded that the milled restorations showed a smaller marginal gap and better internal fit than the conventional material [3]. Both of these studies followed the same technique for measurement of the marginal gap and internal fit by using a replica method that utilized polyvinyl siloxane impression material. These two parameters were measured at nine different points both bucco-lingually and mesiodistally using a microscope at 10× magnification.

Kelvin Khng et al. studied the marginal integrity of milled versus conventional materials. Both the vertical and horizontal marginal integrity in facial and lingual aspects were measured using a polarized light traveling microscope at 4× magnification. The authors reported that the milled interim crown showed a smaller vertical marginal discrepancy (vertical gap at facial—0.13 mm and lingual—0.09 mm; horizontal gap at facial—0.10 mm and lingual—0.02 mm) in comparison to the conventionally fabricated interim crowns. However, no significant difference in the horizontal component was found [29]. Lastly, Yao et al. compared the marginal fit of various conventional and milled materials before and after thermal cycling (TC) using a stereomicroscope. The two conventional materials’ marginal fit values were reported as −0.27 ± 0.04 mm before TC and 0.40 ± 0.06 mm after TC; and −0.28 ± 0.05 mm before TC and −0.51 ± 0.06 mm after TC, respectively. The two milled materials’ marginal fit values were reported as −0.16 ± 0.03 mm before TC and −0.16 ± 0.04 mm after TC; and −0.015 ± 0.03 mm before TC and 0.17 ± 0.03 mm after TC, respectively. This study concluded that one milled material showed a better marginal fit than other materials before and after TC [6].

### 3.5. Mechanical Properties

#### 3.5.1. Surface Roughness

Aldahian et al. compared the surface roughness of both milled and 3D-printed materials with conventional materials. The roughness of the traditional, milled, and 3D-printed provisional materials was observed to be 4.17 μm, 3.28 μm, 5.61 μm, respectively; and the wear was reported to be 17.79 mm^3^, 13.63 mm^3^, and 10.81 mm^3^, respectively. This study concluded that both the milled and 3D-printed materials had less wear compared to conventional ones; however, the 3D-printed material showed the highest surface roughness [38]. The surface roughness was measured with a 3D optical noncontact surface microscope, and the wear calculated was the surface loss value; i.e., the difference between the images taken before cyclic loading and after cyclic loading. Myagmar et al. conducted a study comparing the mechanical properties of 3D-printed resin, milled PMMA, and conventional PMMA materials. The roughness of the 3D-printed material was 0.13 ± 0.01 nm before the chewing simulation, and a roughness of 0.48 ± 0.07 nm and 0.59 ± 0.06 nm was observed after the simulation of 30K and 60K chewing cycles. For the milled PMMA material, the roughness was reported to be 0.19 ± 0.03 nm before simulation and then 0.88 ± 0.05 nm and 1.27 ± 0.49 nm after 30K and 60K simulated chewing cycles, respectively. The conventional PMMA material showed a roughness of 0.26 ± 0.02 nm before the simulation and then a roughness of 0.92 ± 0.10 nm and 1.64 ±0.44 nm after the simulation of 30K and 60K chewing cycles, respectively. A confocal laser scanning microscope was used to analyze the surfaces before and after the chewing simulation. Images were obtained via laser excitation. A scanning electron microscope was used to measure the resultant wear. The study concluded that the chewing simulation tests produced the least effects on the surface roughness of the milled PMMA material [41].

#### 3.5.2. Mechanical Strengths

Ahmadzadeh et al. compared the fracture strength of conventional bis-acryl (1326.6 ± 101.7 N) and PMMA (1179.1 ± 133.5 N) with milled PMMA (1494.3 ± 117.1 N) materials. The authors concluded that the milled PMMA material showed a higher fracture resistance than the conventional materials [30]. Coelho et al. conducted a study comparing the mechanical properties of two conventional and two milled materials with or without a cantilever. The conventional bis-acryl and PMMA materials demonstrated a fracture strength of 1287 N and 1390 N without a cantilever and a fracture strength of 1954 N and 1268 N with a cantilever, respectively. Meanwhile, the milled PMMA materials showed a fracture strength of 3126 N and 3136 N without the cantilever and fracture strengths of 2649 N and 1634 N with the cantilever, respectively. The authors concluded that the milled PMMA materials showed a higher strength compared to the conventional ones [31]. Both of these studies used a universal testing machine to measure the fracture strengths of their samples.

Cakmak et al. evaluated the flexural strength of milled PMMA and conventional interim materials with and without a surface sealant after thermocycling. The study concluded that the flexural strength of the different milled PMMA materials (34.80 ± 6.61, 31.11 ± 6.56 A, and 32.84 ± 7.83 MPa) were higher than those of the conventional bis-acryl resin (15.92 ± 3.95 MPa) and PEMA material (15.79 ± 9.78 MPa) [33]. Jeong et al. compared the fracture toughness of two milled PMMA (3.08 and 2.67 Mpa·m^1/2^ two 2 3D-printed resins (1.50 and 1.52 Mpa·m^1/2^), two conventional bis-acryl (1.60 and 2.00 Mpa·m^1/2^), and two conventional PMMA (1.91 and 1.62 Mpa·m^1/2^) interim materials, and the milled PMMA showed the highest fracture toughness value in comparison to the other materials in the study [34]. Al Deeb et al. compared the failure load and compressive strength of milled PMMA, conventional PMMA, and 3D-printed interim fixed prosthesis materials. The fracture load was measured via application of a static load at a crosshead speed of 1 mm/min until fracture. The authors concluded that the milled PMMA material showed a superior fracture load (687.86 ± 46.72 N) to the conventional PMMA (492.7 ± 61.8 N) and 3D-printed resin materials (534.8 ± 46.1 N). Similarly, the milled PMMA material also showed a superior compressive strength (2.44 ± 0.27 MPa) to the conventional PMMA (1.65 ± 0.20 MPa) and 3D-printed resin materials (1.80 ± 0.15 MPa) [39].

Abdullah et al. conducted a study comparing the fracture strength of three milled PMMA materials and one conventional bis-acryl resin, and the authors concluded that the milled PMMA interim restorations had superior fracture strengths (347 ± 30.71, 375.04 ± 36.97, and 361.52 ± 27.76 N) in comparison to the conventional one (284.9 ± 49.07 N) [40]. In another study, a similar conclusion was drawn by the same researchers: the milled polyetheretherketone (PEEK) (802.23 ± 111.29 N) and milled PMMA materials (361.01 ± 21.61 and 719.24 ± 95.17 N) had superior fracture strengths in comparison to the conventional bis-acryl resin (416.40 ± 69.14 N) [3]. Both the study designs used a static load with a universal testing machine at a crosshead speed of 1 mm/min until the samples fractured.

Karaokutan et al. studied the fracture strength of conventional PMMA (843.71 ± 83.46 and 711.09 ± 179.18 N), polyurethane polymethacrylate (1009.0 ± 84.50 N), bis-acryl resins (1392.1 ± 344.11 and 910.05 ± 77.09 N), and milled PMMA (745.23 ± 94.75 and 1106 ± 134.65 N) materials. Among all the materials, the conventional bis-acryl resins showed higher fracture strengths compared to the other conventional and milled materials [35]. Rayyan et al. compared the mechanical properties of milled and conventional interim FDP materials, including water sorption, wear, hardness, flexural strength, and fracture strength. The milled interim restorations showed a water resorption of 8.7 ± 0.7 mm/mm^3^, wear at 0.0012 wt%, a hardness of 21.2 ± 1 VHN, a flexural strength of 142 ± 12 Mpa, and a fracture strength of 1289 ± 56 N. The authors concluded that the milled interim crowns presented stable physical and mechanical properties and may be used for long-term interim restorations [2]. The cemented samples were sectioned, and stereomicroscopy revealed no dye penetration and no discoloration of the interim cement after 50,000 cycles of thermocycling in all of the samples. Yao et al. compared failure loads of conventional bis-acryl resins and milled PMMA materials before and after thermocycling. One milled PMMA showed higher failure loads both before (124.10 ± 6.45 MPa) and after (95.39 ± 10.48 MPa) thermocycling [6]. Vally et al. compared the compressive strengths of various conventional and milled restorative materials. Among the materials compared, the authors concluded that the conventional bis-acryl resin showed the highest compressive strength (383.64 MPa), which was higher than that of the milled PMMA material (373.44 MPa) [36]. Alt et al. studied the fracture strength of conventional polyethyl methacrylate acrylic (PEMA), bis-acryl resin, and milled PMMA materials at intervals of 1 day, 1 week, and 3 months. The authors concluded that the milled PMMA material showed a higher fracture strength with thermocycling at 1 day, 1 week, and 3 months in comparison to the conventional materials [37]. Yao et al., Alt et al., and Vally et al. utilized a universal testing machine in their studies to determine the fracture loads.

#### 3.5.3. Esthetic Outcomes

Atria et al. conducted a study comparing the translucency parameter of conventional PMMA and bis-acryl resin with milled PMMA and 3D-printed resin interim FDP materials before and after thermocycling. A commercially available spectrophotometer was used to measure the color coordinates from the center of the samples. The study concluded that thermocycling had the least effect on the milled PMMA material for translucency in comparison to the other materials in the study [41]. Rayyan et al. compared the color alteration (ΔE) of milled PMMA and conventional PMMA acrylic, bis-acryl resin, and thermoplastic resin after immersion in coffee, tea, carbonated cola, and red wine. A calibrated dental colorimeter was used in the study. The analysis showed a large degree of ΔE in the conventional PMMA (6.7 ± 2), bis-acryl resin (7.1 ± 1.5), and thermoplastic resin (5.4 ± 3.1) than the milled PMMA material (2.1 ± 0.2). The L values in the manually fabricated specimens showed a significant decrease, whereas a significant increase was noted in the yellow scale (b*). Moreover, these samples had significant surface scratches and air bubbles, which led to further staining. The authors thus concluded that the milled PMMA material showed a better color stability [2].

## 4. Discussion

This systematic review evaluated studies that compared the marginal fit, mechanical properties, and esthetics outcomes between CAD-CAM milled, 3D-printed, and conventional interim FDP materials. Among all the studies published between 2011 and 2021, 19 articles were selected for the systematic review. Among the 19 studies, only a single randomized controlled trial was part of the review, and the remaining 18 were in vitro studies. Of the 19 studies, 16 studies demonstrated better results with CAD-CAM interim FDP materials, while the remaining 3 studies showed better results with conventional materials.

A dental restoration is often regarded as successful when it offers a good marginal and internal fit that can withstand the oral environment [3]. The fit of the restoration is often studied in the cervical area and at the axial and occlusal walls of the tooth preparations. A poor marginal adaptation of interim restoration may result in solubility of the cement, microleakage, secondary caries, and periodontal disease [42]. Sailer et al. reported that 11% of dental abutments have secondary caries after 3 years of clinical performance. An increase of up to 22% in the caries rate was noted by the same authors after 5 years [43].

Many authors have studied the marginal adaptation of traditional fixed prostheses in various in vitro and in vivo study settings [44,45,46,47,48]. A marginal gap below 120 µm is considered clinically acceptable. This marginal gap is essential for the accurate insertion of the fixed prosthesis because it allows an even layer of the luting cement (mean values between 25 and 50 µm) and improves the mechanical strength and retention of the restoration [49].

The marginal fit of an interim restoration depends on several factors such as teeth preparation, impression technique, materials, luting cement, and the technology used in the fabrication of the restoration [27]. In this systematic review, four studies compared the marginal fit of the CAD-CAM interim FDP materials with the conventional ones. In the randomized controlled trial by Cheng et al., provisional crowns fabricated with the milled technology demonstrated a significantly better fit overall than those fabricated using the conventional workflow [26]. Although the marginal fit of the CAD-CAM interim prostheses was better than the conventional ones, the difference was not statistically significant. A study by Angwarawong et al. concluded that both milled and 3D-printed interim crowns had smaller marginal discrepancies than those fabricated with conventional materials [28]. Meanwhile, Kelvin Khng et al. displayed similar results using milled interim restorations [29]. Contrary to these findings, a study by Wu et al. found that conventional interim crowns had a better internal fit and a smaller marginal discrepancy compared to milled and 3D-printed interim crowns [27]. This was also supported by an in vitro study by Mohajeri et al. in which the authors noticed that the mean marginal gap values for temporary crowns fabricated conventionally were the lowest. However, these crowns were fabricated on an implant abutment and not on teeth [50].

Overall, more studies favored the marginal fit of the milled interim FDPs over the conventional ones. This could be explained by the materials utilized in the fabrication of the crowns. CAD-CAM technology uses prefabricated industrially polymerized blocks, whereas conventional techniques use PMMA or composite-based acrylic resins. The conventional materials have the disadvantage of polymerization shrinkage, which is eliminated when a milled prosthesis is used, thereby resulting in a better marginal fit of milled/3D-printed restorations.

In this review, eight studies investigated the mechanical properties of CAD-CAM and conventional interim restorations. In the study by Ahmadzadeh et al., milled PMMA demonstrated a higher fracture resistance in comparison to the conventional bis-acryl resin and PMMA materials [30]. Similarly, studies by Coelho et al. and Alt et al. reported a superior fracture strength of milled interim prostheses compared to conventional ones [31,37]. Jeong et al. studied the effect of various types of surface roughness and repair materials on the shear bond strength and the fracture toughness of conventional and CAD/CAM interim materials [34]. They reported a higher fracture toughness of milled PMMA compared to other conventional and 3D-printed materials [34]. In addition, the shear bond strength values when CAD/CAM materials were repaired chairside were within clinically acceptable limits as compared to the other materials [34].

In contrast, Karaokutan et al. reported a higher fracture strength in conventional materials than in milled PMMA materials [35]. Vally et al. compared the compressive strengths of seven different interim FDP materials and also reported a higher compressive strength of the conventional materials than the other groups (including milled materials) [35]. Both these papers compared only one brand of milled CAD/CAM provisional materials in the in vitro study settings. Hence, their results must be treated with caution. In addition, no 3D-printed materials were a part of these studies. Most of the studies in this systematic review used static loading with a universal testing machine to measure the fracture load of the specimens. This type of testing does not simulate the oral environment, and hence its results cannot be considered a gold standard [35,36].

The superior mechanical properties of milled interim FDP materials could be attributed to the fact that milled PMMA materials are industrially polymerized under optimal manufacturing conditions. These conditions instill better mechanical properties and biocompatibility in the interim restorations [45]. The presence of better mechanical properties also enhances the longevity of the interim restorations [2]. Long-term milled interim prostheses could be ideal for implant treatment, periodontal therapy that requires longer follow-up, and full-mouth rehabilitations in which the prostheses could be exposed to functional loading [7].

In addition, studies by Aldahian et al. and Al Deeb et al. compared both the mechanical properties and marginal fit of both milled and 3D-printed interim restorations with conventional ones [38,39]. Aldahian et al. concluded that 3D-printed interim restorations demonstrated improved fit, adaptation, and wear properties compared to other groups. The surface roughness of the 3D-printed group was highest followed by the conventional and milled PMMA groups [38]. Al Deeb et al. reported that the marginal fit of 3D-printed interim restorations was comparable to that of milled ones [39]. Abdullah et al. (studies in 2016 and 2018) and Yao et al. compared milled interim restorations with conventional ones [3,6,40]. The authors concluded that the milled interim restorations demonstrated smaller marginal discrepancies and better mechanical properties than the conventional restorations.

Surface roughness is known to directly affect the color of the material and the adherence of the plaque, thereby hampering esthetics and causing gingival inflammation [46,47]. The composition of the material is the key factor that determines the surface roughness. The 3D-printed resins used in the study by Atria et al. lacked filler particles, thus leading to a higher surface roughness [41]. They concluded that the milled CAD/CAM materials had superior surface roughness values as compared to the 3D-printed or conventional interim restorations [41]. However, Myagmar et al. compared the wear resistance and surface roughness of interim restorations fabricated using 3D-printing, milling, and conventional interim materials during a chewing simulation [32]. The results of the study demonstrated greater wear resistance with 3D-printed and milled interim materials compared to the conventional material after 60,000 cycles of the chewing simulation [32]. A significantly higher surface roughness was evident in conventional materials when compared to the 3D-printed resins. The Atria study used thermocycling to test the samples, whereas the Myagmar study used a chewing simulator. In addition, different materials were used as study samples for both CAD/CAM and 3D-printed samples. This could probably explain the difference in the results of the two studies [32,41].

Rayyan et al. compared the color stability, water sorption, wear resistance, surface hardness, fracture resistance, and microleakage of milled interim restorations with those of conventional interim restorations [2]. The authors concluded that the milled interim crowns presented stable physical and mechanical properties. In fact, the study by Atria reported that of all the materials tested, the color stability noted for the 3D-printed samples was the lowest. They believed that a wide variety of factors such as the curing time, printing orientation (degree), and post-processing affected the polymerization of the resins, which in turn affected the final properties of the material [41].

Technology for 3D printing is the future of clinical dentistry. It finds wide applications in all dental fields ranging from orthodontics and prosthodontics to maxillofacial surgery. The quick turnaround time offered by this technology is a great boon for both clinicians and patients, especially when used as an interim provisional material for fixed prosthodontics. There is currently a lack of clinical evidence regarding the materials and printers available for 3D printing [13]. It is recommended that the manufacturers state the applications of their material in specific clinical scenarios (such as long-term or short-term provisionalization) or whether the material can be used for a single unit or a multi-unit prosthesis. More in vitro and in vivo studies are required to test the mechanical, optical, and biological properties of these materials to determine their long-term success and survival rates [13].

To our understanding, no other systematic review comparing the properties of CAD-CAM and conventional interim restorations has been published to date. The limitation of this systematic review was that most of the studies analyzed were in vitro studies. In addition, CAD/CAM interim fixed dental prostheses are newer treatment modalities in dentistry with more studies available after 2019, and this systematic review only included 19 studies fitting the inclusion criteria. Various parameters were studied by the authors in different study settings; for example, micro CT was used to assess the marginal gap in some studies [38] (Aldahian et al.), whereas polyvinyl siloxane material was used by others (Abdullah et al.) [3,40]. Hence, there was no standardization, and the results could not be compared. In addition, the influence of clinical parameters such as saliva, occlusal forces/bruxism, the temporary cement used, the length of the prosthesis, a single unit or multi-units, and the skills of the clinician were not considered in most of the publications. Further, clinical and nonclinical studies are essential in dentistry to shed more light on the newer materials and technologies that are being introduced in this field of interim fixed dental materials.

## 5. Conclusions

This systematic review included 19 studies, and only 1 was a randomized clinical trial and provided answers to the predefined focus question on the outcomes of conventional and CAD-CAM interim FDPs. The results suggested that milled interim FPDs offer a better marginal fit, higher mechanical properties, and better esthetic outcomes in terms of color stabilities when compared to the 3D-printed and conventional ones. However, future randomized controlled trials are required to strengthen the existing evidence.

## Figures and Tables

**Table 1:** Focus question and search strategy.

Focus Question	In Natural Teeth, what Are the Outcomes of CAD-CAM Interim FDPs Compared to the Conventionally Manufactured Ones Regarding Marginal Fit, Mechanical Properties, Esthetics, and Color Stability?
Population	1. Teeth [MeSH Terms]: tooth OR dentate OR dentulous. 2. Provisional restoration [MeSH Terms]: temporary dental restoration OR dental restoration, temporary, OR tooth crown, denture, partial, temporary, provisional OR interim and dental restoration OR temporary dental restoration OR provisional crown OR temporary crown OR interim crown OR provisional fixed partial OR interim fixed partial OR temporary fixed partial.
Intervention or Exposure	3. CAD/CAM [MeSH Terms]: computer-aided design [MeSh] OR computer aided manufacturing OR CAD/CAM OR computer dentistry OR computer milled prosthesis OR digital dentistry.
Comparison	4. Conventional provisional restoration [MeSH Terms]: conventional interim restoration OR conventional temporary restoration OR conventional provisional crown OR conventional provisional bridge OR conventional provisional prosthesis OR traditional provisional restoration.
Outcome	5. Marginal fit [MeSH Terms]: Marginal fit OR fit OR gap OR internal OR marginal OR adaptation OR accuracy OR precision OR trueness OR Esthetic OR strength OR superiority OR clinician preference OR clinical efficacy. 6. Mechanical properties [MeSH Terms]: mechanical phenomena OR mechanical processes OR mechanical phenomena OR mechanical processes OR dental restoration wear OR mechanical properties OR fracture toughness OR flexural strength OR surface wear OR compressive strength OR Brittleness OR ductility OR elastic modulus OR fatigue OR hardness OR impact strength OR malleability OR elongation OR proportional limit OR shear strength OR tensile strength OR yield strength OR Young’s modulus. 7. Esthetics [MeSH Terms]: esthetic OR dental esthetic OR cosmetic OR appearance OR color stability OR shade OR value OR hue OR chroma.
Search combination	(1 OR 2 OR 3) AND 4 AND (5 OR 6 OR 7)
Language	English
Electronic databases	PubMed (MEDLINE) Cochrane Central Register of Controlled Trials (CENTRAL) EMBASE Web of Science
Manual journal searches	Journal of Prosthetic Dentistry, Journal of Prosthodontics, International Journal of Prosthodontics, Journal of Dentistry, Journal of the American Dental Association, Journal of Operative Dentistry, personal communications in the New York Academy of Medicine Grey Literature Report, and Dental Materials.
Inclusion criteria	In vitro and in vivo studies Articles published 2000–2022
Exclusion criteria	Case reports with fewer than six patients Studies without comparative methodology Methodology, technique, or review article Multiple publications on the same patient population Non-English language

**Table 2:** Newcastle–Ottawa bias assessment for non-RCT.

	Selection				Comparability	Outcome			
Authors, Year	Was the Study Definition Adequate? (1)	Sample Size (1)	Selection of Controls (1)	Definition of Controls (1)	Comparability of Samples and Controls (2)	Assessment of Outcome (1)	Same Method of Ascertainment for All Samples (1)	Statics (1)	Total Quality Score (9)
Aldahian et al., 2021	1	1		1	1	1	1	1	7
Ahmadzadeh et al., 2021	1	1			2	1	1	1	7
Coelho et al., 2021	1	1	1	1	2	1	1	1	9
Myagmar et al., 2021	1	1		1	1	1	1	1	7
Wu et al., 2021	1	1		1	1	1	1	1	7
Çakmak et al., 2020	1		1	1	2	1	1	1	8
Angwarawong et al., 2020	1	1	1	1	2	1	1	1	9
Atria et al., 2020	1	1	1	1	2	1	1	1	9
Jeong et al., 2019	1	1	1	1	2	1	1	1	9
Aldeeb et al., 2019	1	1		1	1	1	1	1	7
Abdullah et al., 2018	1	1		1	1	1	1	1	7
Abdullah et al., 2016	1	1		1	1	1	1	1	7
Kelvin Khng et al., 2016	1	1	1	1	2	1	1	1	9
Karaokutan et al., 2015	1	1	1	1	2	1	1	1	9
Rayyan et al., 2015	1	1	1	1	2	1	1	1	9
Yao et al., 2014	1	1		1	1	1	1	1	7
Vally et al., 2013	1	1	1	1	2		1	1	8
Alt et al., 2011	1	1	1	1	2	1	1	1	9

**Table 3:** Cochrane bias assessment for RCT.

Author, Year	Sequence Generation	Allocation Concealment	Blinding of Participants and Personnel	Blinding of Outcome Assessment	Final Decision
Cheng, 2021	Low	Low	Low	Low	Low risk of bias

**Table 4:** Summarized results of 19 included studies.

Author	Year	Sample Size	Study Type	Material	Fabrication Method	Marginal (M, G, F, I, A, MIF, IN)	Mechanical Properties (FS, CS, FLS, KC, Wear, Rs, EM, PS, FL)	Esthetics/Color	Comments
Aldahian et al.	2021	30	In Vitro	Jet Tooth Shade (PMMA)	Conventional	(M). (A) 269.94 μm (M.MIF) 395.89 μm	(Rs) 4.17 (Wear) 17.79		Freeprint Temp resin 3D-printed interim (samples showed better fit with the smallest discrepancies (211 μm), adaptation (197.82 μm), and least wear (Rs) (10.81) properties compared to other groups. However, surface roughness was the highest in the 3D-printed samples. (Rs) 3.28.
				Cercon base PMMA blocks	CAD/CAM (milled)	(M). (A) 269.52 μm (MIF) 244.95 μm	(Rs) 3.28 (Wear) 13.63		
				Freeprint Temp resin	CAD/CAM (3D-printed)	(M). (A) 197.82 μm (MIF) 211.87 μm	(Rs) 5.61 (Wear) 10.81		
Ahmadzadeh et al.	2021	30	In Vitro	Protemp 4 (Bis-acryl)	Conventional		(FS) 1326.6 ± 101.7 N		PMMA block showed higher fracture resistance (1494.3 ± 117.1 N) in comparison to the conventional material.
				PMMA	Conventional		(FS) 1179.1 ± 133.5 N		
				PMMA block	CAD/CAM (milled)		(FS) 1494.3 ± 117.1 N		
Coelho et al.	2021	40	In Vitro	Protemp4 (Bis-acryl)	Conventional		(FS) 1287 N (cantilever) 1954 N		Provisional fixed partial prostheses produced by Vita CADTemp (FS) 3136 N, Telio CA PMMA (cantilever) 2649 N (CAD/CAM) had higher strength than those fabricated by traditional chairside polymerization.
				Telio CA (PMMA)	CAD/CAM (milled)		(FS) 3126 N (cantilever) 2649 N		
				Dentalon Plus (PMMA)	Conventional		(FS) 1390 N (cantilever) 1268 N		
				Vita CADTemp (acrylate polymer)	CAD/CAM (milled)		(FS) 3136 N (cantilever) 1634 N		
Myagmar et al.	2021	48	In Vitro	NextDent C&B	CAD/CAM (3D-printed)		(Rs) (before) 0.13 ± 0.01 nm (Rs) (30k c) 0.48 ± 0.07nm (Rs) (60k c) 0.59 ± 0.06nm		The 3D printed resin (NextDent C&B) and milled resin Yamahachi PMMA Disk showed greater wear resistance than the conventional interim resin after simulation of the clinical chewing period equivalent to a duration of 1.5 and 3 months.
				Yamahachi PMMA Disk	CAD/CAM (milled)		(Rs) (before) 0.19 ± 0.03nm (Rs) (30k c) 0.88 ± 0.05nm (Rs) (60k c) 1.27 ± 0.49nm		
				Jet (PMMA)	Conventional		(Rs) (before) 0.26 ± 0.02 nm (Rs) (30k c) 0.92 ± 0.10 nm (Rs) (60k c) 1.64 ± 0.44 nm		
Cheng et al.	2021	40	RCT	PMMA Disk; Yamahachi Dental	CAD/CAM (milled)	(M.F) 1.15 ± 0.37			Interim crowns fabricated with the digital workflow (PMMA Disk; Yamahachi Dental) resulted in smaller gap (MF) 1.15 ± 0.37, which resulted in a better fit then the conventional.
				MMA materials (ALIKE; GC (PMMA)	Conventional	(M.F) 1.50 ± 0.69			
Wu et al.	2021	48	In Vitro	LuxaCrown (bis-acryl)	Conventional	Polyvinyl siloxane-replica method (M. discrepancy absolute) 71.3 ± 64.9 Optical coherence tomographic scanning technique (M. discrepancy absolute) 82.7 ± 65.8			The conventionally fabricated provisional crowns using resin-based composite material LuxaCrown presented a lower gap distance (71.3 ± 64.9, 82.7 ± 65.8), which meant a better internal fit and a smaller absolute marginal discrepancy.
				Lava Ultimate (Nano-ceramic)	CAD/CAM (milled)	Polyvinyl siloxane-replica method (M. discrepancy absolute) 96.9 ± 60.2 Optical coherence tomographic scanning technique (M. discrepancy absolute) 99.6 ± 54.6			
				Dima PrintDenture Teeth	CAD/CAM (3D-printed)	Polyvinyl siloxane-replica method (M. discrepancy absolute) 120.8 ± 70.9 Optical coherence tomographic scanning technique (M. discrepancy absolute) 143.1 ± 39.9			
Çakmak et al.	2020	14	In Vitro	M-PM-Disc (PMMA)	CAD/CAM (milled)		(FL) 34.80 ± 6.61		The flexural strength of milled PMMA-based polymers (M-PM-Disc PMMA) (FL) 34.80 ± 6.61 was higher than the flexural strength of conventional bisacrylate composite resin and PEMA IR materials.
				Polident- (PMMA)	CAD/CAM (milled)		(FL) 31.11 ± 6.56A		
				Telio CA (PMMA)	CAD/CAM (milled)		(FL) 32.84 ± 7.83		
				Acrytemp (Bis-acryl)	Conventional		(FL) 15.92 ± 3.95		
				Bosworth Trim (PEMA)	Conventional		(FL)15.79 ± 9.78B		
Angwarawong et al.	2020	40	In Vitro	Unifast Trad (PMMA)	Conventional	(M.G) (before aging) 85 ± 23 (M.G) (after aging) 114 ± 29			The Brylic Solid and Freeprint Temp groups (CAD/ CAM fabricated interim restorations) showed a smaller marginal gap, which resulted in better marginal adaptability than the Unifast Trad and Protemp 4 groups (conventionally fabricated restorations) both before and after artificial aging.
				Protemp 4 (Bis-acryl)	Conventional	(M.G) (before aging) 88 ± 17 (M.G) (after aging) 109 ± 15			
				Brylic Solid (PMMA)	CAD/CAM (milled)	(M.G) (before aging) 54 ± 8 (M.G) (after aging) 74 ± 9			
				Freeprint Temp	CAD/CAM (3D-printed)	(M.G) (before aging) 56 ± 7 (M.G) (after aging) 71 ± 7			
Atria et al.	2020	40	In Vitro	Acrylic resin (Marche 66 shade (PMMA)	Conventional		1.3 mm Thickness Before (TC) (Rs) 0.22 After (TC) (Rs) 0.31 0.6 mm Thickness Before (TC) (Rs) 0.26 After (TC) (Rs) 0.31	1.3mm Thickness Translucency Before (TC) 9.59(0.10) C* After (TC) 9.97 (0.10) D* 0.6 mm Thickness Translucency Before (TC) 17.70 (0.33) c After (TC) 16.81 (0.15) c	The PMMA milled (TelioCAD) material had the least effect after thermal cycling for both roughness and translucency in comparison to the other materials in the study.
				Protemp (Bis-acryl)	Conventional		1.3 mm Thickness Before (TC) (Rs) 0.18 After (TC) (Rs) 0.23 0.6 mm Thickness Before (TC) (Rs) 0.20 After (TC) (Rs) 0.25	1.3 mm Thickness Translucency Before (TC) 11.07 (0.12) B* After (TC) 10.59 (0.05) C* 0.6 mm Thickness Translucency Before (TC) 17.33 (0.49) c After (TC) 14.76 (0.22) d	
				TelioCAD (PMMA)	CAD/CAM (milled)		1.3 mm Thickness Before (TC) (Rs) 0.20 After (TC) (Rs) 0.19 0.6 mm Thickness Before (TC) (Rs) 0.20 After (TC) (Rs) 0.20	1.3 mm Thickness Translucency Before (TC) 11.75 (0.02) B* After (TC) 12.11 (0.03) B* 0.6 mm Thickness Translucency Before (TC) 20.07 (0.10) b After (TC) 20.59 (0.04) b	
				Raydent C&B for temporary crown and bridge	CAD/CAM (3D-printed)		1.3 mm Thickness (Rs) 0.26 (after TC) 0.54 0.6 mm Thickness Before (TC) (Rs) 0.21 After (TC) (Rs) 0.60	1.3 mm Thickness Translucency Before (TC) 17.51 (0.10) A* After (TC) 14.85 (0.21) A* 0.6 mm Thickness Translucency Before (TC) 24.60 (0.07) a After (TC) 23.19 (0.10) a	
Jeong et al.	2019	80	In Vitro	Nextdent C&B	CAD/CAM (3D-printed)		(KC) 1.5(0.24) MPa·m1/2)		Yamahachi PMMA disk milled material showed the highest fracture toughness value ((KC) 3.08(0.16) Mpa·m1/2) in comparison to the other materials in the study.
				ZMD-1000B Temporary	CAD/CAM (3D-printed))		(KC) 1.52(0.19) MPa·m1/2		
				Yamahachi PMMA disk	CAD/CAM (milled)		(KC) 3.08(0.16) Mpa·m1/2		
				Huge PMMA block	CAD/CAM (milled)		(KC) 2.67(0.12) Mpa·m1/2		
				Jet PMMA	Conventional		(KC) 1.91(0.23) Mpa·m1/2		
				Alike PMMA	Conventional		(KC) 1.62(0.22) Mpa·m1/2		
				Luxatemp (Bis-acryl)	Conventional		(KC) 1.60(0.09) Mpa·m1/2		
				Protemp 4 (Bis-acryl)	Conventional		(KC) 2.00(0.23) Mpa·m1/2		
Al Deeb et al.	2019	30	In Vitro	CAD-CAM blocks Ceramill TEMP, (PMMA)	CAD/CAM (Milled)	(M.MIF) 68.2 ± 18.1 nm	(FL) 687.86 ± 46.72 N (CS) 2.44 ± 0.27 MPa		Ceramill TEMP (PMMA) milled materials showed superior marginal fit, internal adaptation, fracture load and compressive strength than the conventional material.
				TrimPlus, (PMMA)	Conventional	(M.MIF) 283.3 ± 98.6 nm	(FL) 492.7 ± 61.8 N (CS) 1.65 ± 0.20 MPa		
				Form 2, Formlabs, (PMMA)	CAD/CAM (3D-printed)	(M.MIF) 84.7 ± 27.5 nm	(FL) 534.8 ± 46.1 N (CS) 1.80 ± 0.15 MPa		
Abdullah et al.	2018	40	In Vitro	VITA CAD-Temp (Acrylate polymer)	CAD/CAM (milled)	(M.G)59.97 ± 11.1 μm, (IN. F) 117.8 ± 15.58 μm	(FS) 347 ± 30.71 N		The milled PMMA samples showed a smaller marginal gap and internal fit with the smallest ArtBloc^®^ Temp (45.58 ± 9.99 μm, 109.27 ± 19.21 μm); the milled PMMA samples also showed a higher fracture strength with the highest ArtBloc^®^ Temp (375.04 ± 36.97 N); the milled PMMA showed smaller marginal gap and internal fit with a superior fracture strength in comparison to the conventional.
				ArtBloc^®^ Temp (PMMA)	CAD/CAM (milled)	(M.G) 45.58 ± 9.99 μm, (IN. F) 109.27 ± 19.21 μm	(FS) 375.04 ± 36.97 N		
				PMMA DISK	CAD/CAM (milled)	(M.G) 62.19 ± 12.9 μm, (IN.F) 123.16 ± 23.97 μm	(FS) 361.52 ± 27.76 N		
				Acrytemp (Bis-acryl)	Conventional	(M.G) 138.6 ± 10.1 μm (IN. F) 140.1 ± 26.53 μm	(FS) 284.9 ± 49.07 N		
Abdullah et al.	2016	40	In Vitro	VITA CAD-Temp (Acrylate polymer)	CAD/CAM (milled)	(M.G)60.61 ± 9.99 um, (IN.F) 124.94 ± 22.96 um	(FS) 361.01 ± 21.61 N		All of the milled samples showed a smaller marginal gap and internal fit with the smallest marginal gap PEEK (46.75 ± 8.26um), and Telio CAD-Temp had the smallest internal fit (110.95 ± 11.64um); milled samples also showed a higher fracture strength with the highest PEEK (802.23 ± 111.29 N) except vita cap-temp, which was lower than the conventional protemp. Milled materials showed a smaller marginal gap and internal fit with superior fracture strength in comparison to the conventional.
				PEEK	CAD/CAM (milled)	(M.G) 46.75 ± 8.26 um, (IN.F) 113.14 ± 23.55 um	(FS) 802.23 ± 111.29 N		
				Telio CAD-Temp (PMMA)	CAD/CAM (milled)	(M.G) 56.10 ± 5.65 um, (IN.F) 110.95 ± 11.64 um	(FS) 719.24 ± 95.17 N		
				ProtempTM4 (Bis-GMA)	Conventional	(M.G) 193.07 ± 35.96 um, (IN.F) 143.48 ± 26.74 um	(FS) 416.40 ± 69.14 N		
Kelvin Khng et al.	2016	60	In Vitro	Telio CAD-CE (PMMA)	CAD/CAM (milled)	(M.I) (Vertical) (Facial) 0.18 mm (Lingual) 0.09 mm (M.I) (Horizontal) (Facial) 0.18 (0.05) (Lingual) 0.03 mm			The Paradigm MZ100-E4D (milled provisional crown) showed a smaller vertical marginal discrepancy in comparison to the conventionally fabricated crowns. However, there was no significant difference in the horizontal component.
				Paradigm MZ100-E4D (Composite)	CAD/CAM (milled)	(M.I) (V) F 0.13 mm L 0.09 mm (M.I) (H) F 0.10 (0.05) L 0.02 (0.03) mm			
				Caulk (Composite)	Conventional	(M.I) (V) F 0.29 (0.14) L 0.13 (0.07) mm (M.I) (H) F 0.10 (0.08) L 0.16 (0.18) mm			
				Jet (PMMA)	Conventional	(M.I) (V) F 0.15 (0.06) L 0.11 (0.06) mm (M.I) (H) F 0.02 (0.04) mm L 0.13 (0.09) mm			
Karaokutan et al.	2015	60	In Vitro	Imident (PMMA)	Conventional		(FS) 843.71 ± 83.46 N		Structur Premium (bis-acryl) conventional material showed ((FS) 1392.1 ± 344.11 N) a higher fracture strength in comparison to the other groups (including milled materials, which came in second).
				Structur Premium (Bis-acryl)	Conventional		(FS) 1392.1 ± 344.11 N		
				Systemp c&b ll Polyurethane polymethacrylate	Conventional		(FS) 1009.0 ± 84.50 N		
				Acrytemp (Bis-acryl)	Conventional		(FS) 910.05 ± 77.09 N		
				Takilon BBF (PMMA)	Conventional		(FS) 711.09 ± 179.18 N		
				Temdent Classic (PMMA)	CAD/CAM (milled)		(FS) 745.23 ± 94.75 N		
				Cercon Base (PMMA)	CAD/CAM (milled)		(FS) 1106 ± 134.65 N		

Abbreviations: Marginal (M), Gap (G), Fit (F), Integrity (I), Adaptation (A), Misfit (MIF), Internal (IN), Fracture strength (FS), Compressive strength (CS), Flexural strength (FLS), Fracture toughness (KC), Roughness (Rs), Elastic modulus (EM), Peak stress (PS), Failure load (FL), Change in color (E), Water sorption (WS), Thermal cycling (TC).

## Data Availability

The data presented in this study are available in the tables and figure within the article.

## Supplementary Materials

The following supporting information can be downloaded at: https://www.mdpi.com/article/10.3390/ma16051996/s1, Table S1: PRISMA checklist.
